# Cardioprotective effects of short-term empagliflozin treatment in db/db mice

**DOI:** 10.1038/s41598-020-76698-8

**Published:** 2020-11-12

**Authors:** Bernhard Radlinger, Florian Hornsteiner, Sabrina Folie, Willi Salvenmoser, Bernhard J. Haubner, Thomas Schuetz, Simone Haas, Claudia Ress, Timon E. Adolph, Karin Salzmann, Bernhard Weiss, Herbert Tilg, Susanne Kaser

**Affiliations:** 1 Department of Internal Medicine I, Christian Doppler Laboratory for Metabolic Crosstalk, Medical University Innsbruck, Anichstraße 35, 6020 Innsbruck, Austria; 2 Department of Internal Medicine I, Medical University Innsbruck, Innsbruck, Austria; 3 Insitute of Zoology and Center of Molecular Biosciences Innsbruck (CBMI), Leopold Franzens University Innsbruck, Innsbruck, Austria; 4 Department of Internal Medicine III, Medical University Innsbruck, Innsbruck, Austria

**Keywords:** Cardiology, Endocrinology, Pathogenesis

## Abstract

Sodium glucose transporter (SGLT)-2 inhibitors have consistently shown cardioprotective effects independent of the glycemic status of treated patients. In this study we aimed to investigate underlying mechanisms of short-term empagliflozin treatment in a mouse model of type II diabetes. Male db/db mice were fed a western type diet with or without enrichment with empagliflozin for 7 days. While glucose tolerance was significantly improved in empagliflozin treated mice, body weight and fasting insulin levels were comparable in both groups. Cardiac insulin signaling activity indicated by reduced proteinkinase B (AKT) phosphorylation was significantly decreased in the empagliflozin treated group. Remarkably, mitochondrial mass estimated by citrate synthase activity was significantly elevated in empagliflozin treated mice. Accordingly, mitochondrial morphology was significantly altered upon treatment with empagliflozin as analysed by transmission electron microscopy. Additionally, short-term empagliflozin therapy was associated with a changed cardiac tissue cytokine expression in favor of an anti-inflammatory pattern. Our data suggest that early cardioprotection in empagliflozin treated mice is independent of a reduction in body weight or hyperinsulinemia. Ameliorated mitochondrial ultrastructure, attenuated cardiac insulin signaling and diminished cardiac inflammation might contribute to the cardioprotective effects of empagliflozin.

## Introduction

Selective inhibitors of SGLT-2 such as empagliflozin were found to exert unexpected cardio- and nephroprotective effects in clinical trials and thus have become important therapeutic agents in type II diabetes, especially in patients with preexisting cardiac or renal disease. SGLT-2 mediates glucose reabsorption in the proximal tubular system and inhibition of the transporter induces glucosuria that is associated with a reduction in blood glucose levels and a moderate decrease in body weight^1^. In the EMPA-REG outcome trial empagliflozin treatment resulted in an early reduction of hospitalization rate for heart failure^2^. Cardiovascular outcome trials with SGLT-2-Inhibitors and especially the DAPA-HF trial show beneficial effects also in non-diabetics suggesting glucose independent effects^2–5^. The underlying mechanisms are not fully understood, however, altered ventricular loading conditions due to increased natriuresis and a fuel shift towards ketone body utilization caused by a changed insulin-to-glucagon ratio are discussed to explain early cardioprotective effects.

Although atherosclerosis and traditional risk factors are very commonly found in type II diabetics, high prevalence of heart failure in these patients cannot solely be explained by macrovascular disease. Instead, type II diabetes confers a more than twofold increased risk for development of heart failure even after adjusting for increased macrovascular risk^6,7^. Diabetic cardiomyopathy is now recognized as its own entity and evidence shows that it is highly prevalent in type II diabetes patients^8,9^. Even though heart failure is associated with states of insulin resistance like type II diabetes, the role of cardiac tissue insulin signaling in the context of heart failure is not clear. Recent data suggest that the cardiac Insulin/PI3K/AKT signaling pathway may contribute to cardiac dysfunction^10^. Furthermore, cardiomyocyte mitochondrial dysfunction was found in several animal models of diabetic cardiomyopathy^11^.

Aim of this study was to investigate systemic and cardiac tissue specific effects of empagliflozin treatment that are independent on known pleiotropic effects like reduction in body weight, change in body composition and improved hyperinsulinemia of long-term therapy^12–14^. To do so, we studied cardiac effects of short-term empagliflozin treatment in leptin-receptor dysfunctional (db/db) mice, a model for type II diabetes and diabetic cardiomyopathy^15^.

## Results

### Short-term EMPA treatment effects on glucose metabolism and body weight

**Figure 1:**
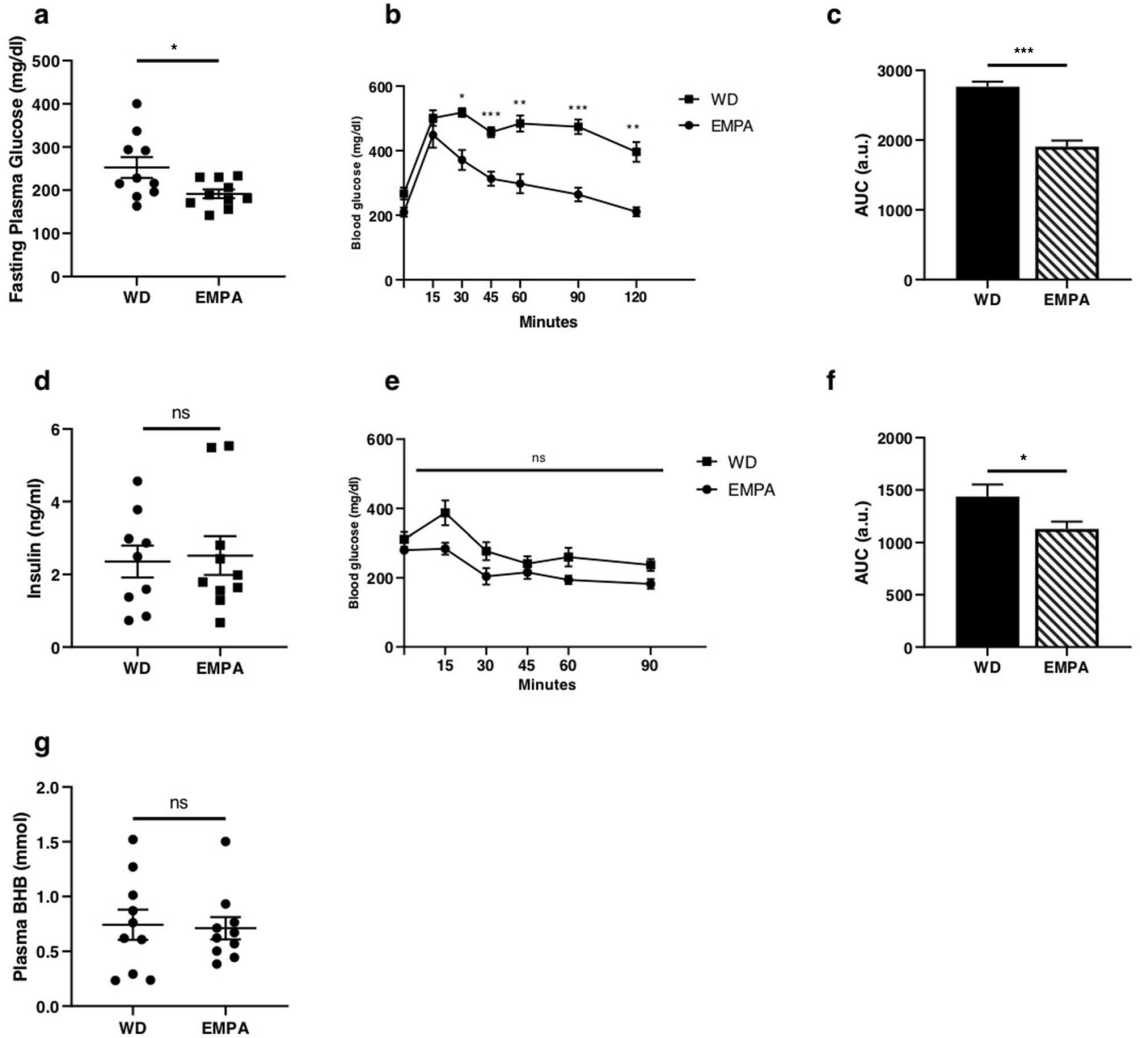
Short-term EMPA treatment leads to a changed glucose metabolism (**a**) Fasting glucose and (**b**) oral glucose tolerance test (oGTT) after a 4 h fast with 1 g glucose/ kg body weight (*n* = 8–10). (**c**) AUC of oral glucose tolerance test. (**d**) Fasting insulin and (**e**) intraperitoneal (i.p.) insulin tolerance test (ipITT) after a 4 h fast with 1U insulin/kg body weight (*n* = 8–9). (**f**) AUC of intraperitoneal insulin tolerance test. (**g**) Fasting beta hydroxy butyrate (BHB) levels (*n* = 9–10). Data are expressed as means ± SEM, *p < 0.05, **p < 0.01, ***p < 0.001, *n.s.* = not significant. WD, Western type diet only; EMPA, Western type diet enriched with empagliflozin.

7–8 weeks old male db/db mice were fed a Western type diet with or without empagliflozin added to the diet. After 1 week, bodyweight and food intake of EMPA treated db/db mice were similar between the groups (Table 1). Fasting glucose levels and oral glucose tolerance determined by an oral glucose tolerance test were significantly improved in mice treated with empagliflozin (Fig. 1a–c). In contrast, fasting insulin levels and insulin sensitivity estimated by intraperitoneal insulin tolerance test was comparable between the groups (Fig. 1d,e). However, when data from intraperitoneal insulin tolerance test were analyzed as AUC, insulin sensitivity was slightly but significantly increased in empagliflozin treated mice (Fig. 1f). Additionally, beta-hydroxybutyrate (BHB) concentrations (Fig. 1g) and blood count was comparable between groups (Table 1).

**Table 1:** Characteristics of animals at baseline and after 7 days. Mean corpuscular volume (MCV) and Mean corpuscular haemoglobin (MCH). Data are expressed as means ± SEM, n = 10.

	WD	EMPA	*p * value
Bodyweight (g) (Baseline)	32.9 ± 0.8	32.6 ± 1.1	0.777
Bodyweight (g) (1 week)	40.2 ± 0.6	38.9 ± 0.9	0.244
Food Intake (g/day)	7.0 ± 0.3	7.6 ± 0.5	0.362
White blood cells (10^3^/µl)	7.43 ± 0.92	7.46 ± 1.42	0.986
Granulocytes (10^3^/µl)	0.84 ± 0.25	0.73 ± 0.33	0.794
Erythrocytes (10^6^/µl)	7.26 ± 0.19	7.24 ± 0.15	0.932
Hemoglobin (g/dl)	13.59 ± 0.31	13.19 ± 0.20	0.286
Hematocrit (%)	34.8 ± 1.04	34.0 ± 0.77	0.539
MCV (fl)	47.94 ± 0.30	46.99 ± 0.35	0.053
MCH (pg)	18.74 ± 0.19	18.25 ± 0.15	0.057

### Empagliflozin treatment affects mitochondrogenesis and mitochondrial ultrastructure

**Figure 2:**
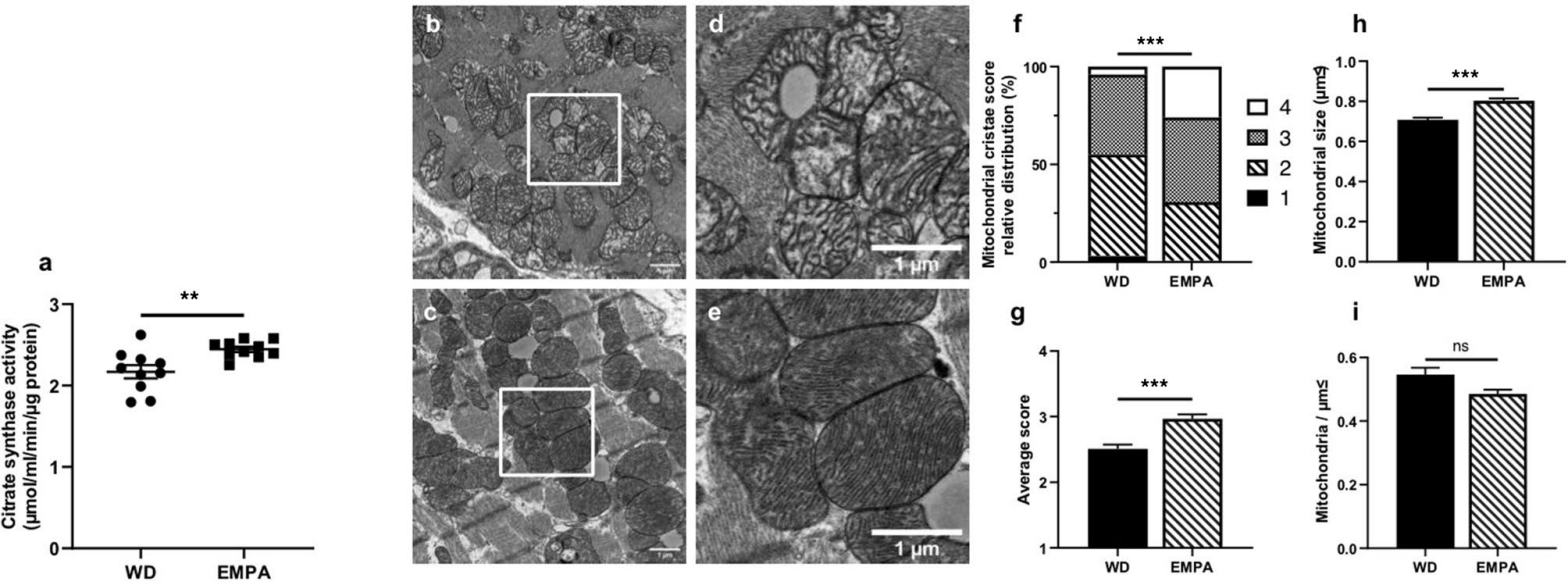
EMPA treatment induces changes in mitochondrial mass, morphology and size (**a**) Citrate synthase activity was measured using a photometric kinetic assay (*n* = 10). Representative transmission electron microscopy (TEM) images of left ventricular cardiac tissue of untreated (**b** and **d**) and EMPA treated mice (**c** and **e**). Scale bar = 1 µm, *n* = 3. Relative distribution (**f**) and average score (**g**) of mitochondrial cristae score (see Supplementary Fig. 2 for details). (**h**) Average size in µm^2^ of a single mitochondrium. (**i**) mitochondrial density as expressed in mitochondria per µm^2^. Data are expressed as means ± SEM, ***p < 0.001, **p < 0.01, *n.s.* = not significant. WD, Western type diet only; EMPA, Western type diet enriched with empagliflozin.

Citrate synthase activity which is required for the first step of the TCA cycle and reflects a measure of mitochondrial mass was significantly increased in EMPA treated mice (Fig. 2a). Mitochondrial ultrastructure was analyzed using transmission electron microscopy (TEM). Remarkably, mitochondrial cristae appearance was different in EMPA treated and untreated mice. Representative images of observed structural changes are shown in Fig. 2b–e. Mitochondrial cristae of EMPA treated mice seemed more regularly spaced with a narrow matrix and mitochondria seemed to be more densely packed with cristae compared to untreated mice. Accordingly, mitochondrial cristae score, which was assessed in a blinded fashion, was significantly increased in EMPA treated mice (see Supplementary Fig. 2 for a detailed description of the used cristae score) (Fig. 2f,g). Additionally, average mitochondrial size was larger in empagliflozin treated mice while mitochondrial density remained unchanged (Fig. 2h,i).

Other markers of mitochondrogenesis, such as peroxisome proliferator activated receptor γ coactivator—1α (Pgc1-⍺) and cytochrome c oxidase subunit 8b (Cox8b) mRNA levels as well as cytochrome c oxidase subunit 4 (COX4) protein levels, were unaltered between the two groups (Supplementary Fig. 2a,b). To check if the mitochondrial fusion/fission process is involved, we checked mitochondrial fission factor (MFF) activation via phosphosite Ser146, however there was no significant change between treatment groups (Supplementary Fig. 3c).

### Empagliflozin treatment alters cardiac insulin signaling

**Figure 3:**
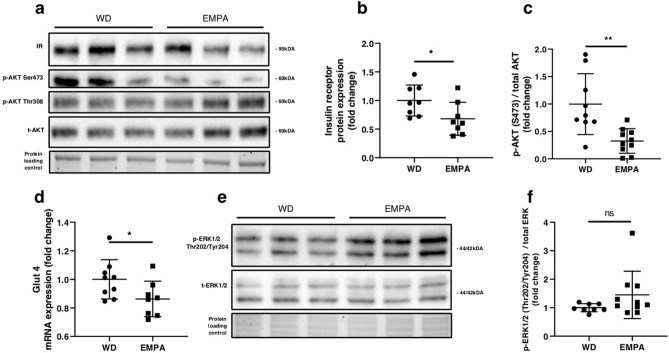
Short-term treatment with EMPA affects cardiac insulin signaling (**a**) Representative Western blot analyses of insulin receptor (IR) phospho-AKT Serine 473, phospho-AKT Thr308 and total AKT. (**b**) Densitometry of IR western blot. (**c**) Ratio of p-AKT Ser473 over total AKT. (**d**) Glut 4 mRNA expression. (**e**) Representative Western blots of phospho-ERK1/2 Thr202/Tyr204 and total ERK1/2 with (**f**) respective ratio of phospho-ERK1/2 over total ERK. Data are expressed as means ± SEM, *n* = 8–10, *p < 0.05, **p < 0.01, *n.s.* = not significant. Full-length images of cropped gels/blots are available in Supplementary Fig. 3a,b. WD, Western type diet only; EMPA, Western type diet enriched with empagliflozin.

As cardiac insulin signaling may contribute to diabetic cardiomyopathy we analyzed the activity of the cardiac insulin signaling pathway. Short term EMPA treatment was associated with a significant downregulation of insulin receptor protein expression (Fig. 3a,b). AKT phosphorylation at phosphosite Ser473 was significantly decreased in empagliflozin treated mice while phosphorylation at phosphosite Thr308 remained unchanged (Fig. 3a,c). Additionally, Glut4 mRNA expression was significantly reduced upon EMPA treatment (Fig. 3d). Phosphorylation of Extracellular-signal Regulated kinase 1/2 (ERK1/2) was not affected by empagliflozin treatment (Fig. 3e,f).

### Short-term EMPA treatment influences cardiac tissue cytokine expression and regulators of fibrosis

**Figure 4:**
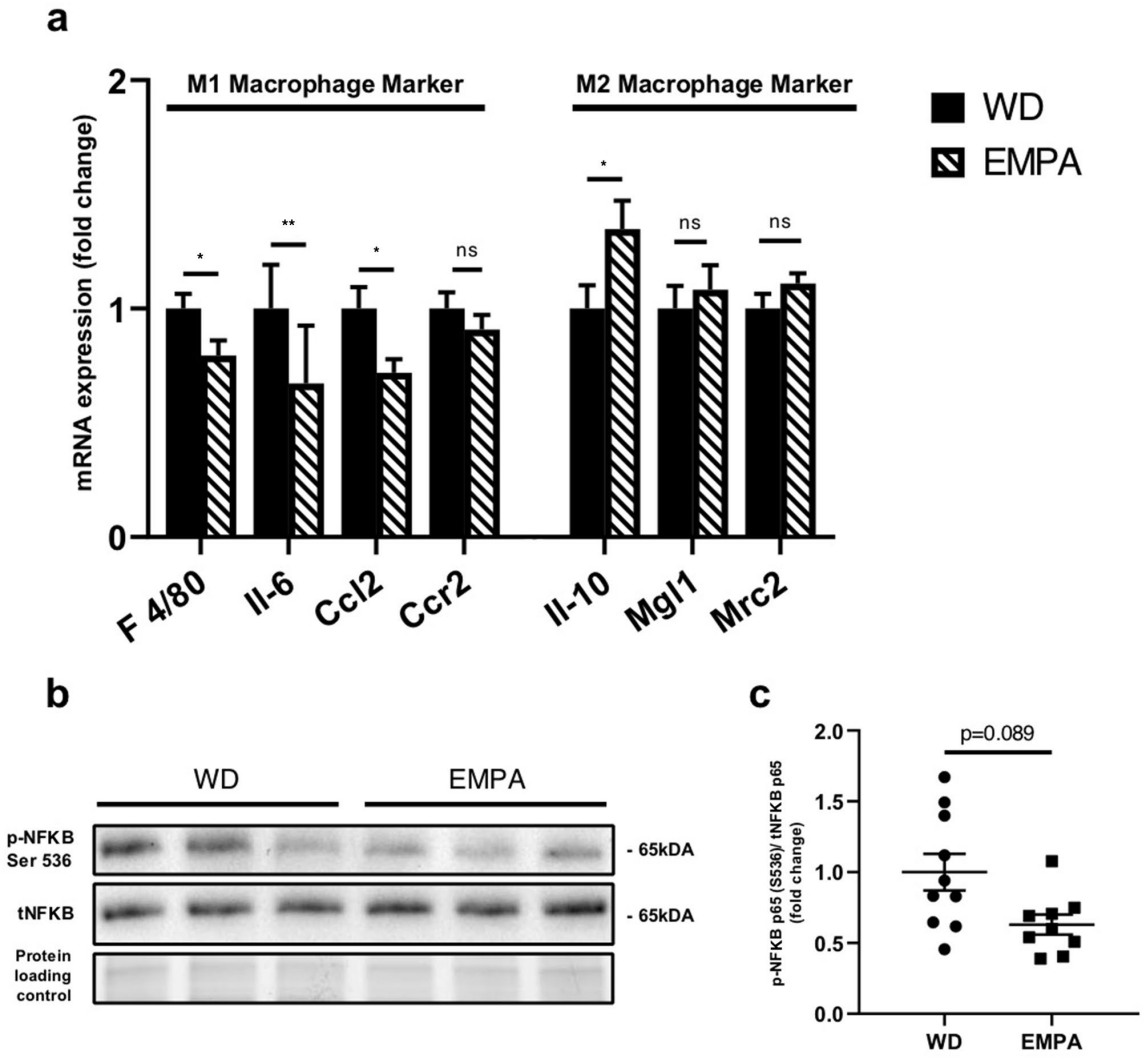
EMPA treatment affects cardiac tissue cytokine expression (**a**) mRNA expression of EGF-like module-containing mucin-like hormone receptor-like 1 (F 4/80), Interleukin-6 (Il-6), Chemokine ligand 2 (Ccl2), chemokine receptor type 2 (Ccr2), Interleukin-10 (Il-10), macrophage galactose-type lectin-1 (Mgl1) and C-type mannose receptor 2 (Mrc2). (**b**) Representative western blot of phospho-NF-κB Ser536 and total NF-κB with respective ratio (**c**) of phospho-NF-κB Ser 536 over total NF-κB. Data are expressed as means ± SEM, *n* = 8–10, *p < 0.05, **p < 0.01, *n.s.* = not significant. Full-length images of cropped gels/blots are available in Supplementary Fig. 4. WD, Western type diet only; EMPA, Western type diet enriched with empagliflozin.

Markers of predominantly pro-inflammatory M1 polarized macrophages including EGF-like module-containing mucin-like hormone receptor-like 1 (F4/80), Interleukin-6 (Il-6), Chemokine ligand 2 (Ccl2) and Chemokine receptor 2 (Ccr2) were downregulated in cardiac tissue of empagliflozin treated mice while Interleukin-10 (Il-10) reflecting the more anti-inflammatory M2 phenotype was upregulated on mRNA level (Fig. 4a). Macrophage galactose-type lectin-1 (Mgl1), C-type mannose receptor 2 (Mrc2) mRNA levels and phosphorylation of activating nuclear factor of activated B-cells (NfKB) were comparable between groups. (Fig. 4b,c). Additionally, RECK (reversion-inducing cysteine-rich protein with Kazal motif) a regulator of cardiac fibrosis was found to be significantly upregulated in EMPA treated mice (Supplementary Fig. 6).

## Discussion

Diabetic cardiomyopathy is commonly found in patients with type II diabetes and is associated with increased mortality^16,17^. SGLT-2 inhibitors have consistently shown to reduce hospitalization rates for heart failure^2–4^ and are known for their beneficial pleiotropic systemic effects besides induction of glycosuria. These systemic effects include a moderate reduction in weight, natriuresis and consecutive hemodynamic changes, improved glucose tolerance and amelioration of hyperinsulinemia^18^. The underlying mechanisms of their cardioprotective effects are still unkown.

In this study we performed a short-term experiment with empagliflozin treatment in db/db mice in order to control for long-term pleiotropic effects including weight reduction and improvement of hyperinsulinemia. We did this to study both systemic effects and cardiac tissue specific effects of empagliflozin treatment in a mouse model of obesity and type II diabetes, prone to heart failure at an older age^19^. We were especially interested in investigating whether pathways or markers that are known to be critically involved in the pathophysiology of diabetic cardiomyopathy are affected by empagliflozin therapy. These include a shift in myocardial fuel metabolism, mitochondrial dysfunction, hyperactivation of cardiac insulin signalling and cardiac inflammation.

Regarding tissue specific effects, Baartscheer and colleagues^20^ recently reported that empagliflozin directly inhibits Na^+^/H^+^ exchanger (NHE) activity in cardiomyocytes thereby increasing mitochondrial Ca2 + concentrations suggesting that empagliflozin might directly affect mitochondrial function in cardiomyocytes. Another study investigating cardiac tissue specific action of empagliflozin showed reduced stiffness in isolated cardiac muscle fibres of human and murine origin^21^.

In the past, a shift in myocardial metabolism from fat and glucose oxidation towards ketone bodies has been discussed to explain improved heart function^22^. This hypothesis was based on the finding of increased plasma β-hydroxybutyrate (BHB) concentrations resulting from reduced portal insulin-to-glucagon ratio and consequently increased lipolysis in empagliflozin treated patients^23^. However, in our study BHB concentrations were similar in empagliflozin treated and control mice suggesting that short-term beneficial effects of empagliflozin on cardiac function are not primarily due to a fuel shift from glucose and lipid oxidation towards myocardial ketone body metabolism in db/db mice.

Regarding cardiac function we performed echocardiography on a subset of mice (Supplementary Fig. 1 and Supplementary Table 1) suggesting trends towards increased ejection fraction (EF) and decreased left ventricular end diastolic diameter (LVEDd) in EMPA treated mice. Results are limited by sample size, because due to the lack of previous experiments with empagliflozin and echocardiography in db/db mice our study was underpowered.

Impaired mitochondrial function is a hallmark of diabetic cardiomyopathy^6,24^. In humans, altered mitochondrial bioenergetics have been demonstrated by reduced phosphocreatine to ATP ratio^25–27^. In animal models of diabetic cardiomyopathy, like the db/db mouse model^15^, altered mitochondrial ultrastructure and impaired mitochondrial respiration that ultimately are associated with impaired cardiac function like reduced ejection fraction have been reported previously^6,28,29^.

The effect of SGLT-2 inhibition on mitochondrial fusion and fission dynamics has been documented in rat models of ischemia–reperfusion injury / myocardial infarction previously^30,31^. In our study phosphorylation of MFF, a key regulator of mitochondrial fission^32^, was unchanged between groups, however we used a shorter treatment period and a different animal model compared to previous studies.

While expression levels of mitochondrial biogenesis marker Pgc1-⍺ were unaffected by short-term empagliflozin, citrate synthase activity of the cardiac tissue was significantly increased in empagliflozin treated mice. Citrate synthase activity typically serves as a marker of mitochondrial mass^33^. Ultrastructure of mitochondria of cardiomyocytes, as shown by transmission electron microscopy, was profoundly altered in EMPA treated mice compared to untreated animals. Mitochondrial cristae were more regularly spaced and densely packed compared to quite irregularly shaped cristae with enlarged mitochondrial matrix in control mice. Although not directly tested in this study, alterations in mitochondrial cristae ultrastructure were associated with impaired mitochondrial respiration previously^34^.

Additionally, TEM analysis revealed that mitochondrial density was comparable between the groups but the size of mitochondria of cardiomyocytes was significantly greater in empagliflozin treated mice. Although changes in mitochondrial size and count have been published before^30^, our study adds the change in mitochondrial ultrastructural morphology and used a considerably different model.

Taken together, our findings of increased citrate synthase activity, changed mitochondrial size and morphology suggest that short-term empagliflozin treatment is associated with improved mitochondrial function and might explain the rise in cardiac ATP production as seen in ex vivo perfused hearts of EMPA treated db/db mice^35^.

Upregulation of the Insulin/PI3K/AKT pathway is characteristically found in an early state of diabetic cardiomyopathy^10,36^. In our study we found that phosphorylation of AKT at phosphosite Serine 473 was significantly diminished. Our results showing decreased insulin receptor protein expression, reduced AKT phosphorylation and decreased Glut4 mRNA expression in empagliflozin treated mice suggest that cardiac insulin signaling activity is decreased in empagliflozin treated mice. Typically, reduced insulin signaling is a hallmark of insulin resistance. However, in the setting of diabetic cardiomyopathy it has been shown that upregulation of the Insulin/PI3K/AKT pathway contributes to development of pathological hypertrophy and reduced systolic function^36^. Genetically engineered mice with a reduction of cardiac IR protein expression displayed an improved response to chronic pressure overload with transverse aortic constriction^10^. AKT itself seems to be a central mediator of detrimental mTOR dependent angiogenetic growth signals, as chronic induced overexpression leads to pathological hypertrophy^37^. In a previous study transgenic overactivation of AKT resulted in impaired mitochondrial respiration and decreased mitochondrial fatty acid oxidation suggesting a causal relationship between hyperactivation of cardiac insulin signalling and mitochondrial respiration^38^. Comparable systemic insulin concentrations in empagliflozin treated and control mice further suggest that these effects do not result from improved systemic hyperinsulinemia. Thus we hypothesize that short-term empagliflozin significantly improves over-activation of cardiac tissue insulin signaling activity which contributes to overall cardioprotective effects.

In our study the role of slightly but not significantly increased phosphorylation of ERK1/2 is less clear. On the one hand ERK1/2 activity is thought to be involved in development of cardiac hypertrophy while on the other hand ERK1/2 signaling might have beneficial effects on abnormal healing and cardiac dysfunction via regulation of Fibroblast growth factor 21 (FGF21) signaling^39^.

Both, local and systemic inflammation has been shown to contribute to the development of diabetic cardiomyopathy^40^. Pro-inflammatory M1 macrophages typically occur in early stages of cardiac dysfunction^41^ while typically anti-inflammatory M2 macrophages are associated with diminished inflammation in the heart^42^. Here we interpret the changed cytokine expression pattern as an indicator of a changed cardiac tissue macrophage polarization. These data suggest a shift in polarization from a M1 towards a more anti-inflammatory/pro-reparative M2 phenotype.

Interestingly, we found altered RECK protein expression in hearts of EMPA treated mice. RECK was reported to be downregulated in an animal model of cardiac fibrosis, additionally, in vitro overexpression of RECK showed inhibitory effects on extensive cardiac fibroblast migration^43^. Interestingly, for renal proximal tubular cells (PTECs)^44^ and in kidneys of db/db mice treated with empagliflozin^45^, is has been shown recently that empagliflozin prevents hyperglycemia induced RECK suppression. To the best of our knowledge we are first to show in vivo data on RECK expression in murine cardiac tissue with an SGLT-2 inhibitor. Further studies are warranted to test the effects of empagliflozin treatment on cardiac fibrosis.

Taken together we hypothesize that empagliflozin treatment is associated with improvements of mitochondrial dysfunction, cardiac insulin signaling and inflammation. While body weight and fasting insulin levels were comparable between the groups, empagliflozin treatment was associated with significantly improved glucose tolerance and fasting blood glucose levels. Thus, it cannot be excluded with certainty, that ameliorated glycemia might contribute to reported beneficial effects, however, clinical observations from cardiovascular outcome trials deem this unlikely^2–5^. These trials showed SGLT-2 inhibition to be equally effective in the treatment of heart failure in both patients with and without type II diabetes^5^. Another recent study with experimentally induced myocardial ischemia in non-diabetic pigs also showed blood-glucose independent effects of empagliflozin^46^.

With respect to other known pleiotropic effects of empagliflozin, blood pressure which is moderately decreased in patients treated with SGLT-2 inhibitors^2^ was not influenced by empagliflozin treatment in db/db mice previously^45^, making it unlikely that beneficial effects demonstrated in our study are related to blood pressure lowering capabilities of empagliflozin.

In summary, we show that early cardioprotective effects of empagliflozin treatment in db/db mice cannot be explained by well-known pleiotropic effects of SGLT-2 inhibitors such as reduction of body weight or improved hyperinsulinemia. Mechanistically, improved mitochondrial ultrastructure, ameliorated over-activation of insulin signaling activity and changed macrophage polarization might contribute to cardioprotective effects of empagliflozin.

## Methods

### Animals

6–7 week old male db/db mice (Charles River Laboratories, Sulzfeld, Germany) were kept on standard conditions including a 12/12 h light dark cycle, 23 + /- 2 °C and controlled humidity. Before the start of the experiment mice were allowed to recover from transportation for one week. 24 mice were fed western type diet alone (TD88137 mod., SSNIFF Spezialdiäten GmbH, Soest, Germany) and 24 mice were fed a western type diet (TD88137 mod., SSNIFF) enriched with 30 mg/kg body weight empagliflozin (Boehringer Ingelheim RCV, Vienna, Austria) ad libitum for 7 days. Out of 24 mice per group, 10 were used to obtain tissue and blood samples after the treatment period, 9 were used for oGTT and ipITT and 5 were used for echocardiography totaling in 48 mice used in this study. Mice were assigned to groups randomly. Animals and food were weighed every second day.

After 7 days of treatment, oGTT and ipITT were performed on separate days after a 4 h fast on 9 mice per group. For oGTT 1 g glucose/kg body weight was orally gavaged and for ipITT 1U/kg body weight insulin was injected intraperitoneally. Blood glucose was measured via tail vein puncture and handheld glucometers (Accu-Chek Performa Nano, Roche, Basel, Switzerland) at given timepoints for both tests. Between the two tests animals were allowed to recover for 3 days.

To obtain tissue samples, mice were anaesthetized and sacrificed via cervical dislocation. Blood was taken and subsequently analyzed with a hemocytometer (Animal blood counter, Lab Technologies, Vienna, Austria). Cardiac tissue was either fixed for transmission electron microscopy or snap frozen in liquid nitrogen for later protein and RNA isolation. Tissue samples were taken from 10 mice per group.

All animal procedures were performed in accordance with the guidelines of the Austrian Animal Testing Act of 1988. Approval for this animal study was granted by the Austrian Federal Ministry for Education, Science and Research (Application number *BMWF-66.011/0058/ − V/3b/2018*).

### Transmission electron microscopy

Immediately after harvesting tissue samples from the left ventricle of the heart were fixed overnight in 2,5% glutaraldehyde in 0.1 M cacodylate buffer, pH = 7.4 (CB). Samples were washed with CB before post fixation with 1% osmium tetroxide in 0.05 M CB for 2 h. Again samples were washed with CB before dehydration with an ascending acetone series. Samples were embedded in EMbed 812 resin (Electron Microscopy Sciences, Hatfield, PA, USA) and polymerized at 60 °C for 48 h.

Ultrathin Sects. (70 nm) were cut with an Ultracut S microtome (Leica, Austria), mounted on grids and stained with lead citrate for 2 min. Sections were then examined with a Carl Zeiss Libra 120 transmission electron microscope (Carl Zeiss, Oberkochen, Germany). Images were acquired with a 2 × 2 k high speed camera and ImageSP software (Tröndle, Germany). Magnification of the images was 3200X and 6300X. Tissue samples were available from 3 mice per group. An area of 1500 µm^2^ for each control mouse and 2000 µm^2^ for each EMPA treated mouse was analyzed and scored using Fiji/ImageJ^47^. Altogether more than 4000 mitochondria were scored. Proper implementation of a mitochondrial cristae score was ensured with blinding the data to the treatment for analysis. In short, the appearance of mitochondrial cristae ultrastructure was scored from 1–4. The detailed description of the score which is adapted from Eisner et al.^48^ is shown in Supplementary Fig. 1.

### Citrate synthase activity

Citrate synthase (CS) activity was measured using a commercially available kit (Sigma-Aldrich, St. Louis, MO, USA). In short, oxaloacetate and acetyl-CoA were added to protein samples, the enzymatic activity of CS led to the formation of citrate and unbound CoA. The free SH groups of CoA were photometrically measured at 512 nm every 10 s for 1.5 min using DTNB/TNB. Baseline activity was measured for 1.5 min before the addition of oxaloacetate and afterwards subtracted. Activity readings were measured in triplicates and were normalized with total protein content.

### Echocardiography

Anesthesia was induced with 4% isoflurane and mice were placed on a temperature controlled warming pad. During transthoracic echocardiography anesthesia was maintained with 1.5% isoflurane. A 30‐MHz linear array transducer (Vevo2100 Imaging System, VisualSonics Inc, Toronto, Canada) was used to acquire B-mode and M-mode tracings in the long-axis view of the left ventricle. Ejection fraction was calculated using the LV-trace method in long-axis B-mode tracings and averaged over multiple standard cardiac cycles.

Echocardiography was performed on 5 mice per group but due to insufficient image quality one mouse of the empagliflozin group had to be excluded from further analysis.

### qPCR and immunoblotting

For qPCR analysis RNA was isolated from snap frozen tissue using Quiazol (Qiagen, Venlo, Netherlands) with chloroform. 1 µg RNA was converted to cDNA using High Capacity cDNA Reverse Transcription Kit (Applied Biosystems, Foster City, CA, USA) adding RNase Inhibitor (Applied Biosystems). qPCR was performed on a 7900 HT Fast Real-Time cycler (Applied Biosystems). For a list of used primers/probes see Supplementary Table 2. Hprt was used for normalization of qPCR data.

Protein lysates were obtained from snap frozen tissue samples using a rotor–stator tissue homogenizer and lysis buffer (40 mM KCL, 25 mM Tris, 1% Triton X) with added protease/phosphatase inhibitor (CellSignaling, Danvers, MA, USA). Protein concentration was measured photometrically using a BCA kit (Thermo Scientific, Rockford, IL, USA). For protein quantification 20–30 µg of protein samples were run on 4–20% gradient PA stainfree gels (BioRad, Hercules, CA, USA) and transferred to a nitrocellulose membrane (Amersham, GE Healthcare Bio-Sciences Corp., Piscataway, NJ, USA). *Stainfree* technology (BioRad) enables total protein quantification after gel electrophoresis, which was used for normalization of densitometry results afterwards^49^. After blocking for 1 h, membranes were cut and incubated over night at 4 °C with antibodies of corresponding size. For a list of used antibodies see Supplementary Table 3. Anti-rabbit HRP linked secondary antibody (Jackson ImmunoResearch, West Baltimore Pike, PA, USA) and chemiluminescent ECL (Amersham) was used for visualization of antibody binding. For western blot quantification ImageLab (BioRad) was used.

### Insulin and beta-hydroxybutyrate

Plasma insulin levels were measured using a mouse insulin ELISA kit from CrystalChem (Elk Grove Village, IL, USA). For the measurement of beta-hydroxybutyrate a colorimetric assay kit from Cayman Chemical (Ann Arbor, MI, USA) was used.

### Statistical analysis

All data was checked for normal distribution with Kolmogorov–Smirnov’s test. For parametric data unpaired two-tailed t-test was used and Mann–Whitney test was used accordingly for non-parametric data. For the analysis of oGTT and ipITT repeated measures ANOVA with Bonferroni post-hoc test was used. Additionally AUCs for the oGTT and ipITT were calculated using the linear trapezoidal method. A Chi-quadrat test was used for the relative distribution of mitochondrial cristae score. Statistical significance was conferred at a *p * value < 0.05. Data are expressed as means ± SEM. GraphPad Prism Version 8.4.1 for windows (GraphPad Software, SanDiego, CA, USA) was used for statistical analysis and generation of figures.

## Supplementary information

Supplementary Information.
