# Supplementary material for: Cardioprotective effects of short-term empagliflozin treatment in db/db mice
Source: Sci Rep. 2020 Nov 12;10:19686. doi: 10.1038/s41598-020-76698-8 (PMC7665199; doi:10.1038/s41598-020-76698-8)
Supplement: Supplementary file 1 — Supplementary Information. [file 41598_2020_76698_MOESM1_ESM.pdf]

Supplementary Information

Cardioprotective Effects of Short-Term Empagliflozin Treatment in *db/db* Mice

Bernhard Radlinger[1,2†], Florian Hornsteiner[1,2†], Sabrina Folie[1,2], Willi Salvenmoser[3], Bernhard J. Haubner[4], Thomas Schuetz[4], Simone Haas[1,2], Claudia Ress[1,2], Timon E. Adolph[2], Karin Salzmann[1,2], Bernhard Weiss[1,2], Herbert Tilg[2], Susanne Kaser[1,2*]

1 Christian Doppler Laboratory for Metabolic Crosstalk, Medical University Innsbruck, Innsbruck, Austria

2 Department of Internal Medicine I, Medical University Innsbruck, Innsbruck, Austria

3 Insitute of Zoology and Center of Molecular Biosciences Innsbruck (CBMI), Leopold Franzens University Innsbruck, Austria

4 Department of Internal Medicine III, Medical University Innsbruck, Innsbruck, Austria

[†]authors contributed equally

E-Mail addresses of authors

Bernhard Radlinger – Bernhard.Radlinger@i-med.ac.at;
Florian Hornsteiner – F.Hornsteiner@student.uibk.ac.at;
Sabrina Folie – Sabrina.Folie@i-med.ac.at;
Willi Salvenmoser – Willi.Salvenmoser@uibk.ac.at;
Bernhard J. Haubner – Bernhard.Haubner@tirol-kliniken.at;
Thomas Schuetz – Thomas.Schuetz@i-med.ac.at;
Simone Haas – csat3260@student.uibk.ac.at;
Claudia Ress – Claudia.Ress@i-med.ac.at;
Timon E.Adolph – Timon-Erik.Adolph@i-med.ac.at;
Karin Salzmann – Karin.Salzmann@tirol-kliniken.at;
Bernhard Weiss – Bernhard.Weiss@student.uibk.ac.at;
Herbert Tilg – Herbert.Tilg@i-med.ac.at;
Susanne Kaser – Sanne.Kaser@i-med.ac.at;

***Corresponding author:**

Susanne Kaser
Medical University Innsbruck, Department of Internal Medicine I, Christian Doppler Laboratory for Metabolic Crosstalk
Anichstraße 35, 6020 Innsbruck, AUSTRIA

Telephone Number: 0043 512 504 28536
E-Mail: Susanne.Kaser@i-med.ac.at

SI Figure 1,

Representative images from echocardiography. (a) left parasternal long-axis B-mode and (b) M-mode image of an untreated mouse. (c) left parasternal long-axis B-mode and (d) M-mode image of an EMPA treated mouse.

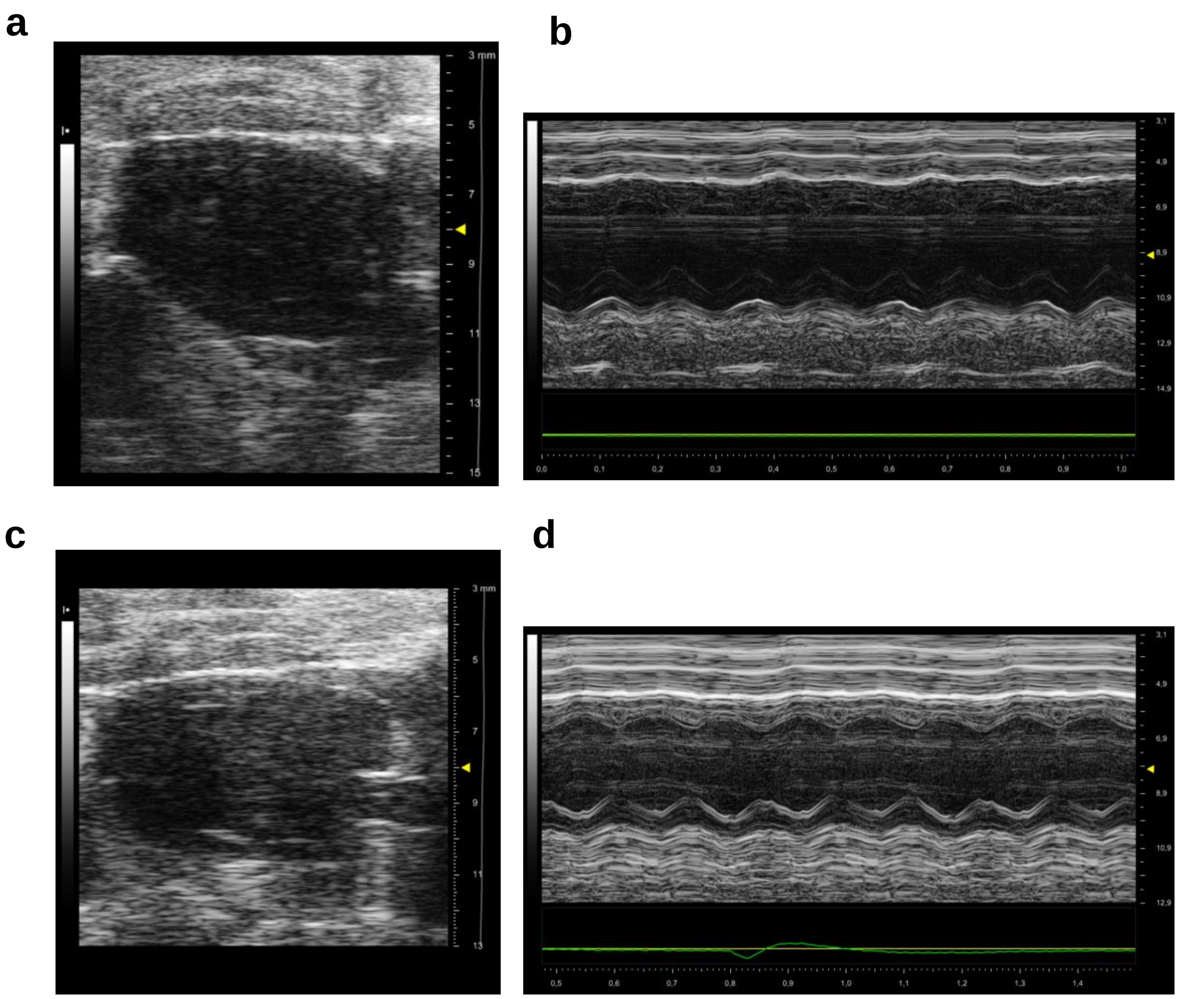

SI Figure 2,

Mitochondrial Cristae Score

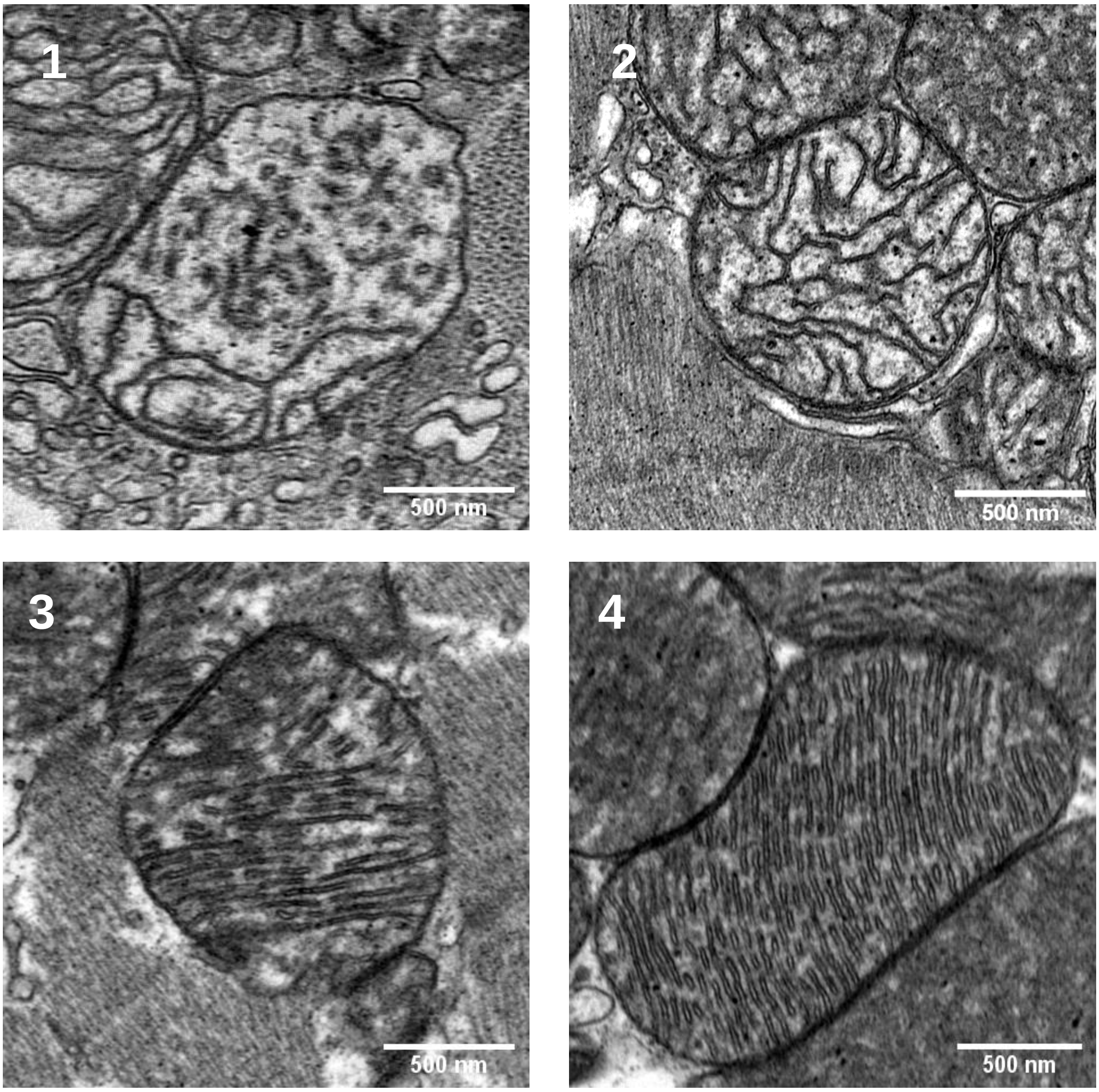

Mitochondrial Cristae Score (1-4)

1 - distorted, swollen looking mitochondria, no clear cristae structure
2 - rounded irregular cristae, widened mitochondrial matrix
3 - clearly visible cristae structure, white spots and some areas of widened matrix
4 - well defined, regular cristae, mitochondria look densely packed with cristae

adapted from Eisner et al., PNAS 2017

SI Figure 3,

Effects of short-term empagliflozin on the expression of Pgc-1α, Cox 8b and Cox IV. (a) Pgc-1α and Cox8b mRNA expression (n=8-9). (b) COX IV protein expression (n=8). (c) p-MFF Ser146 and MFF Protein expression (n=10). Data are expressed as means ± SEM, n.s. = not significant. Full-length images of cropped gels/blots are available in SI Figure 7. WD, Western type diet only; EMPA, Western type diet enriched with empagliflozin.

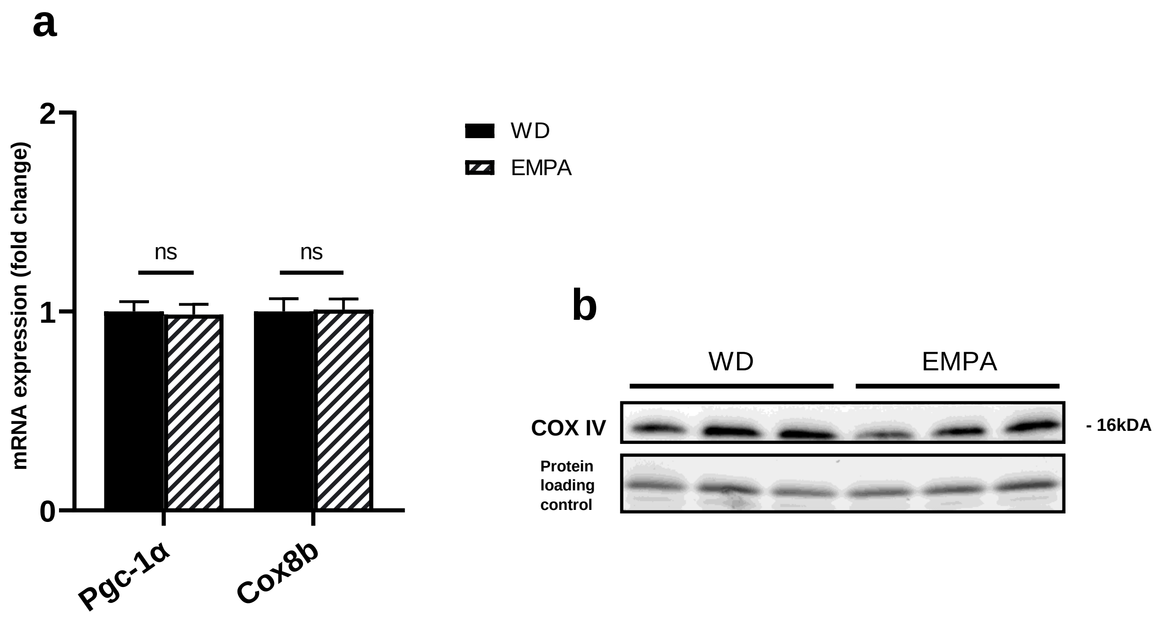

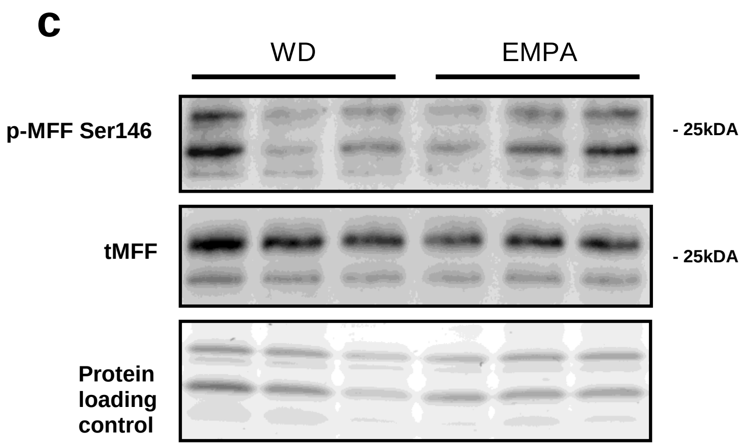

SI Figure 4a,

Full-length Western Blots of Figure 3

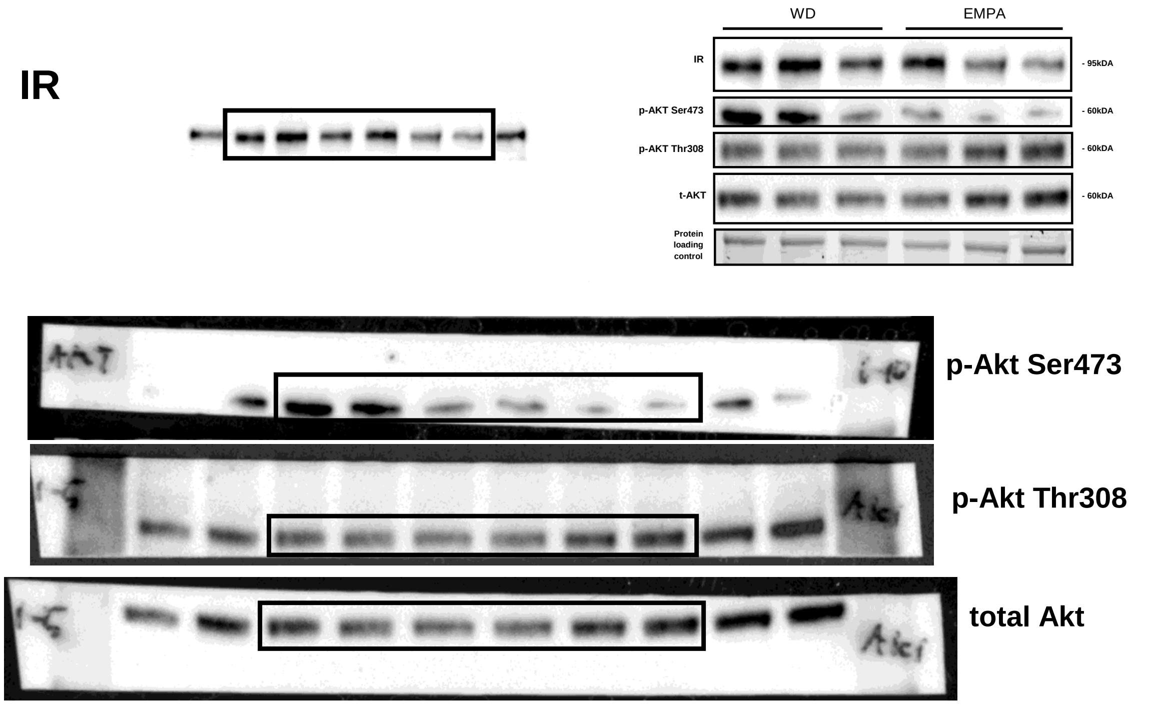

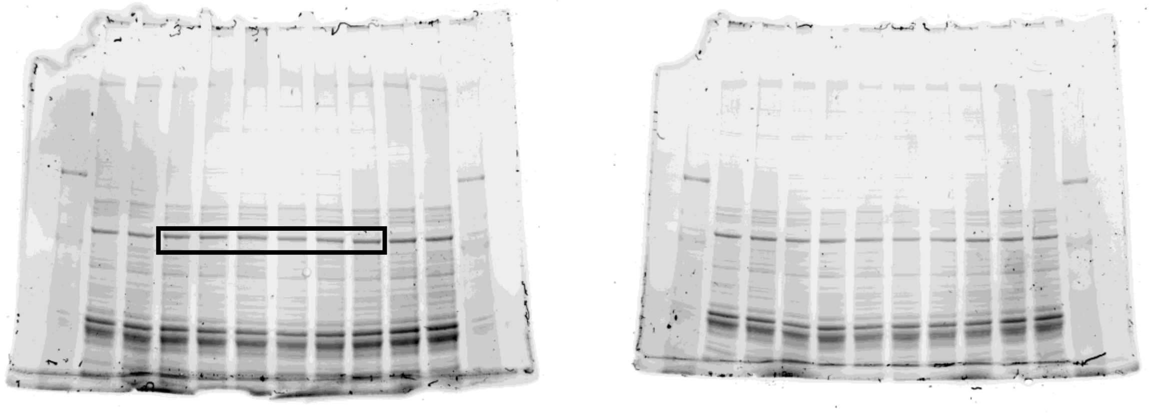

Stainfree Loading Control Replicates 1-5

Stainfree Loading Control Replicates 6-10

SI Figure 4b,

Full-length Western Blots of Figure 3

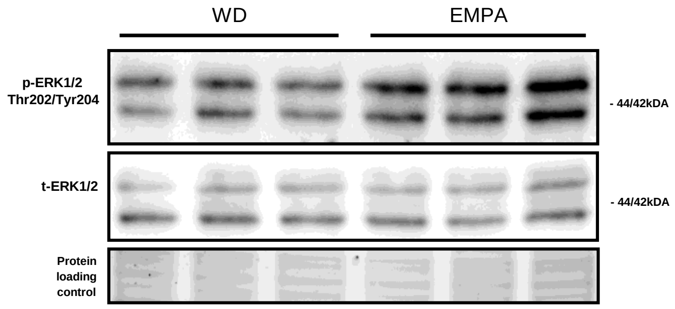

p-Erk 1/2

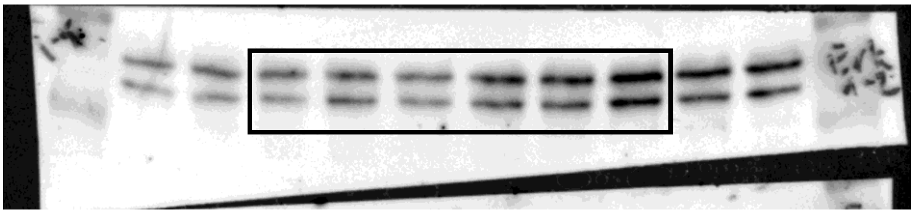

t-Erk 1/2

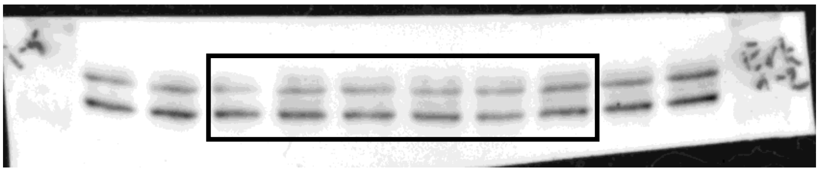

Stainfree
Loading Control

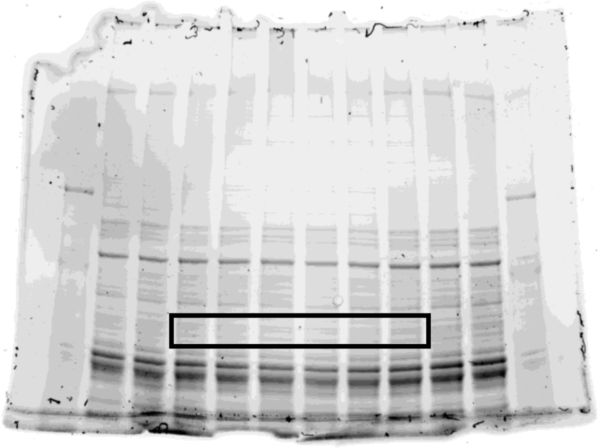

SI Figure 5,

Full-length Western Blots of Figure 4

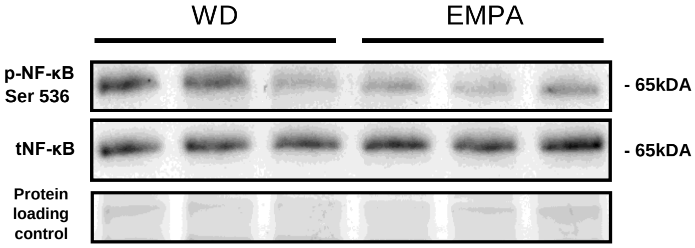

p-NF-κB

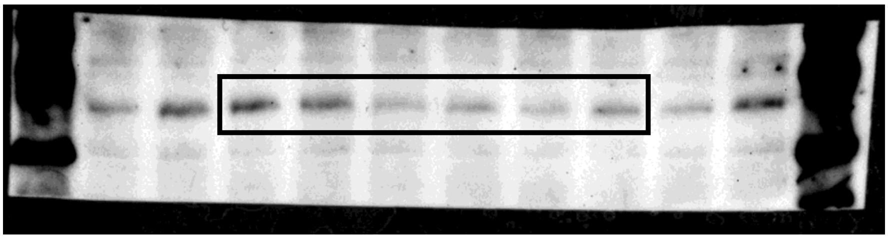

t-NF-κB

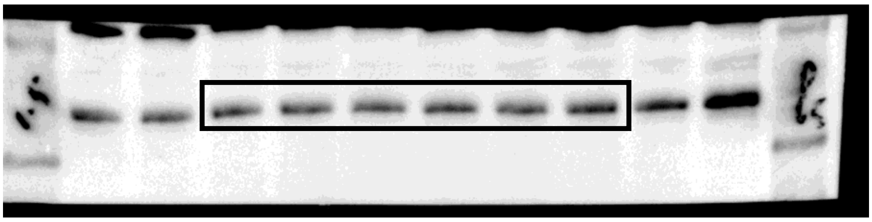

Stainfree
Loading Control

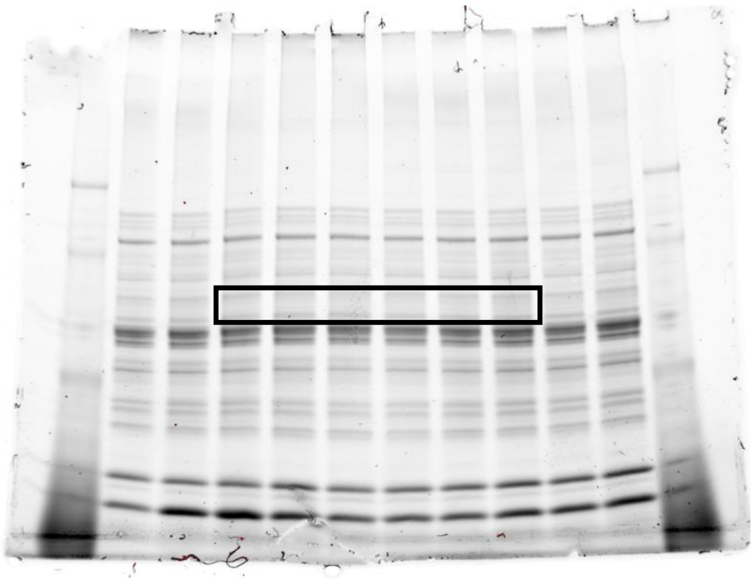

SI Figure 6,

Effects of short-term empagliflozin on RECK expression. (**a**) Representative Western Blot. (**b**) quantification of Western Blot. n=10. Data are expressed as means ± SEM, n.s. = not significant. Full-length images of cropped gels/blots are available in SI Figure 8. WD, Western type diet only; EMPA, Western type diet enriched with empagliflozin.

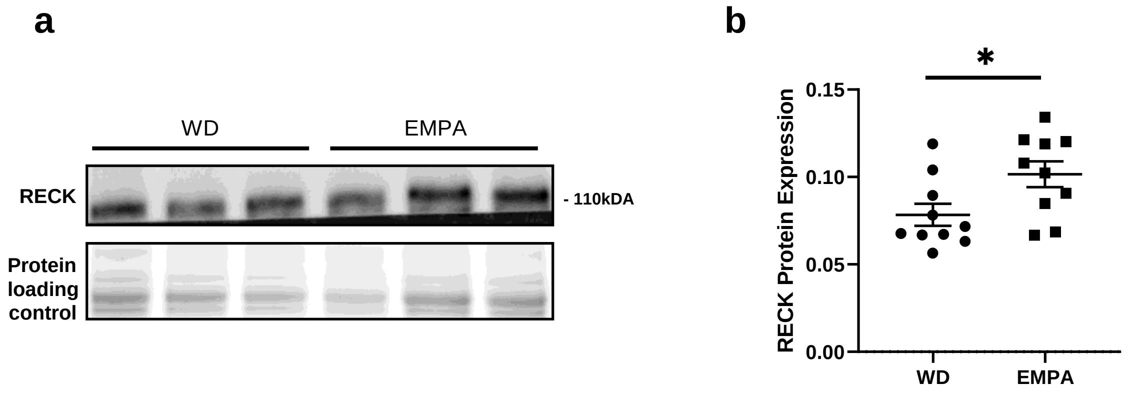

SI Figure 7,

Full-length Western Blots of SI Figure 3

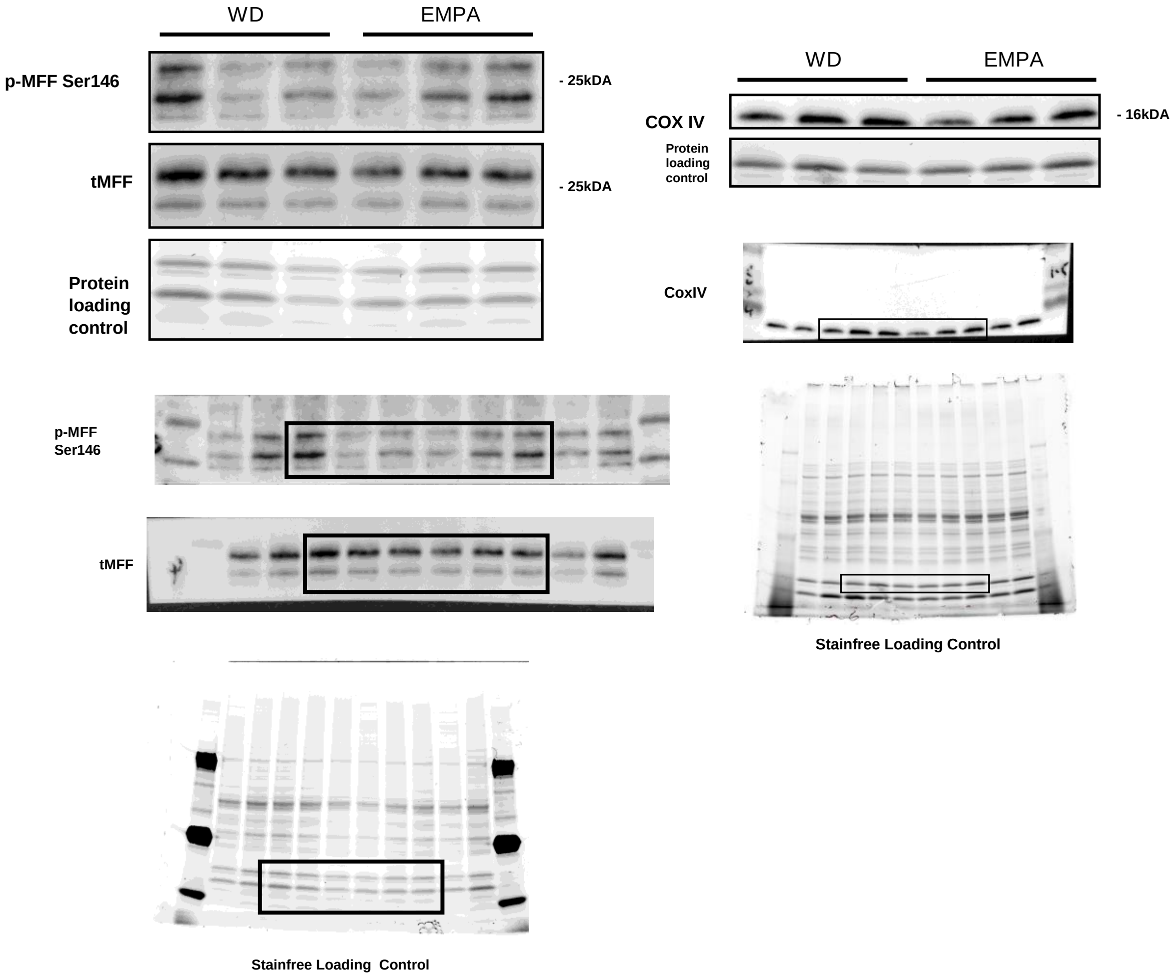

SI Figure 8,

Full-length Western Blots of SI Figure 6

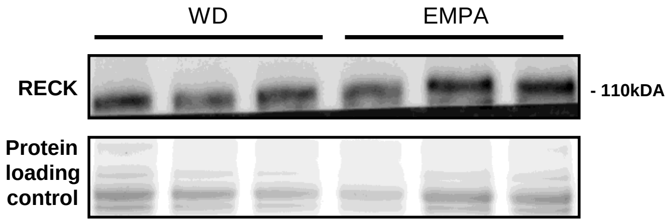

RECK

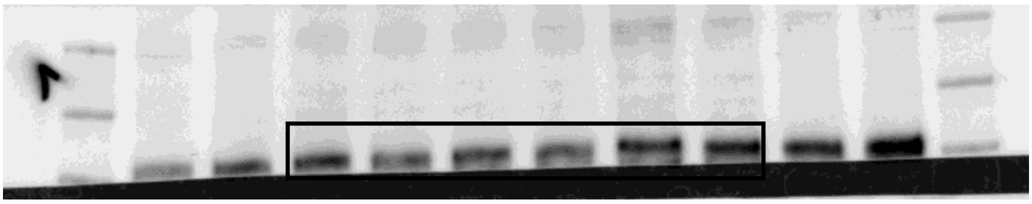

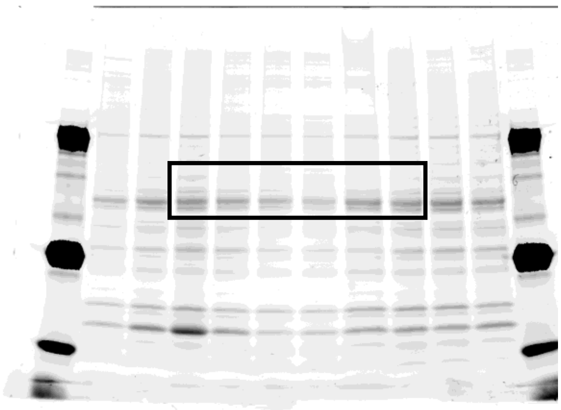

Stainfree Laoding Control

SI Table 1, Echocardiographic assessment of empagliflozin treated and control mice.

Ejection fraction (EF), Fractional shortening (FS), Left ventricular end diastolic diameter (LVEDd), Left ventricular end systolic diameter (LVESd), Heart rate and Left ventricular mass (LV mass). Data are expressed as mean±SEM, n=4-5.

	WD	EMPA	p-value
EF (%)	45.43±2.34	48.69±2.45	0.427
FS (%)	36.69±2.84	40.39±3.04	0.405
LVEDd (mm)	3.95±0.11	3.65±0.14	0.125
LVESd (mm)	2.50±0.12	2.17±0.11	0.09
Heart rate (bpm)	428.4±26.65	445.8±27.22	0.865
LV mass (mg)	114.2±9.90	101.3±13.04	0.446

SI Table 2, Primer sequences used for qPCR

Gene	5’ sequence	3’ sequence
**F 4/80**	CTTTGGCTATGGGCTTCCAGT	GCAAGGAGGACAGAGTTTATCGTG
**Ccl2**	AGCTGTAGTTTTTGTCACCAAGC	GTGCTGAAGACCTTAGGGCA
**Ccr2**	ATTCTCCACACCCTGTTTCG	GATTCCTGGAAGGTGGTCAA
**IL10**	GCTCTTACTGACTGGCATGAG	CGCAGCTCTAGGAGCATGTG
**Mgl1**	TGAGAAAGGCTTTAAGAACTGGG	GACCACCTGTAGTGATGTGGG
**Mrc2**	TACAGCTCCACGCTATGGATT	CACTCTCCCAGTTGAGGTACT

Gene	5’ sequence	3’ sequence	Probe
**Glut4**	CTCATGGGCCTAGCCAATG	GGGCGATTTCTCCCACATAC	CATTGGCGCCTACTCAGGGCTAACATC
**IL-6**	TaqMan Gene Expression Assays 4331182 (Apllied Biosystems)		

SI Table 3, Antibodies used for Western Blotting

Target		Used Dilution
**Insulin Receptor beta**	CellSignaling	1:1000
**AKT**	CellSignaling	1:1000
**Phospho-AKT (Ser473)**	CellSignaling	1:1000
**Phospho-AKT (Thr308)**	CellSignaling	1:500
**ERK1/2 (p44/42)**	CellSignaling	1:1000
**Phospho ERK1/2 (Thr202/Tyr204)**	CellSignaling	1:2000
**NF-κB p65**	CellSignaling	1:1000
**Phospho-NF-κB p65 (Ser536)**	CellSignaling	1:1000
**Phospho-MFF (Ser146)**	CellSignaling	1:1000
**MFF**	CellSignaling	1:1000
**RECK**	CellSignaling	1:1000
**COX IV**	Abcam	1:2000
